# Supplementary material for: Inferring ongoing cancer evolution from single tumour biopsies using synthetic supervised learning
Source: PLoS Comput Biol. 2022 Apr 28;18(4):e1010007. doi: 10.1371/journal.pcbi.1010007 (PMC9049314; doi:10.1371/journal.pcbi.1010007)
Supplement: S1 Text — (PDF) [file pcbi.1010007.s001.pdf]

Supplementary for **"Inferring ongoing cancer evolution from single tumour biopsies using synthetic supervised learning"**

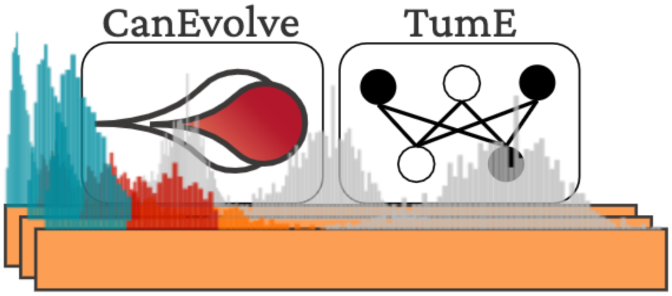

Authors: Tom W. Ouellette[1,2*] and Philip Awadalla[1,2*]

Affiliations:

[1]Ontario Institute for Cancer Research, Department of Computational Biology, Toronto, Ontario M5G 0A3, Canada

[2]Department of Molecular Genetics, Temerty Faculty of Medicine, University of Toronto, Toronto, Ontario M5S 1A8, Canada

*Correspondence to: tom.ouellette@oicr.on.ca or philip.awadalla@oicr.on.ca

Reproducibility and Usage Notes:

- A recommended interactive electronic version of this supplementary can be found at https://tomouellette.gitlab.io/ouellette_awadalla_2021/
- A Julia package for generating synthetic tumour VAF distributions and restructured data for deep learning can be found at https://github.com/tomouellette/CanEvolve.jl
- A python package for cancer evolution inference using convolutional neural networks with Monte Carlo dropout can be found at https://github.com/tomouellette/TumE
- A Zenodo repository containing code, figures, and data used for re-running analyses can be found at https://doi.org/10.5281/zenodo.5931436

1. Description of synthetic tumour generation method

1.1 Pseudo-algorithms for generating synthetic tumours

As outlined in Methods, we implemented two alternative approaches for generating synthetic frequency distributions that recapitulate either positive selection or neutral evolution. The algorithm for simulating tumours (variant allele frequency distributions) subject to positive selection (adapted from Williams et al. 2018 [1]) is outlined in Algorithm 1. The algorithm for generating neutral frequency distributions is outlined in Algorithm 2 (inspired by Caravagna et al. 2020 [2]). The complete algorithm for the paired simulation of positively selected and neutrally evolving tumours is outlined in Algorithm 3. Software to generate synthetic tumours/VAF distributions can be found on GitHub @tomouellette/CanEvolve.jl

Algorithm 1 Tumours subject to positive selection (adapted from Williams et al. 2018)

Simulate tumour with Q positively selected subclones with frequencies $> L$ and $<$ U
while (U $<$ subclone frequency $<$ L) and (number of subclones **not** Q) **do**
  1. Initialize cell with n_{clonal} clonal mutations
  while current population size $< N$ **do**
    2. Randomly sample a cell j
    3. Draw a random number r from Uniform(a, b) where $a = 0$ and $b = b_{max} + d_{max}$ (maximum birth and death rates of all cells in population)
    4. With r, cell j will divide with probability proportional to its birth rate b_j and die with a probability proportional to its death rate d_j
    if $b_j > r$ **then**
      5a. Cell divides and both daughter cells acquire k mutations where k is Poisson distributed with mean equal to the per genome division mutation rate μ
      5i. Each mutation has a probability P_d of being a positively selected driver and initiating a new subclone
      5ii. If the mutation is a driver, it is assigned a selection coefficient s randomly sampled from an exponential distribution with a scale parameter $1/\lambda$
      5iii. The time (in current population size n divided by final population size N) is recorded for every mutation
    else if $b_j + d_j > r >= b_j$ **then**
      5b. Cell dies
    else
      5c. Nothing happens
    end if
  end while
end while
6. Virtual biopsy synthetic tumour and add sequencing noise
7. Remove mutations below hard alternate read cutoff (e.g 2 / mean sequencing depth)

Algorithm 2 Neutrally evolving tumours (inspired by Caravagna et al. 2020)

Simulate a neutral variant allele frequency distribution observed in bulk sequenced tumour populations
1. Randomly sample or set shape α and scale β parameters for a Pareto distribution
2. Generate neutral 'tail' mutations by sampling $n_{non-clonal}$ mutations from Pareto(α, β)
3. Add n_{clonal} heterozygote mutations at a frequency of 0.5
4. With some probability P_{trim}, remove variants below a randomly sampled frequency f (e.g. 0.1 - 0.3) to mimic the loss of neutral tails observed in empirical samples (in general, $P_{trim} < 0.1$)
5. Add sequencing noise
6. Remove mutations below hard alternate read cutoff (e.g 2 / mean sequencing depth)

Algorithm 3 Paired simulation of VAF distributions from neutrally evolving and positively selected tumours

1. Specify number of subclones Q and minimum and maximum subclone frequencies L and U
2. Randomly sample simulation parameters: μ (per genome per division mutation rate), P_d (probability of driver/subclone event), n_{clonal}, $n_{drivers}$, λ^{-1} (scale parameter), mean sequencing depth, ρ (sequencing overdispersion parameter)
3. Run Algorithm 1 until synthetic data is generated with Q subclones at frequencies $<U$ and $>L$
4. Count the approximate number of clonal n_{clonal} and non-clonal $n_{non-clonal}$ mutations in the positive selection scenario
5. Run Algorithm 2 using n_{clonal} * ψ_a and $n_{non-clonal}$ * ψ_b mutations. ψ_a and ψ_b are uniformly sampled numbers that scale the number of clonal and non-clonal mutations to capture additional heterogeneity in training sets.

References

1. Williams MJ, Werner B, Heide T, Curtis C, Barnes CP, Sottoriva A, et al. Quantification of subclonal selection in cancer from bulk sequencing data. Nat Genet. 2018 Jun;50(6):895–903.
2. Caravagna G, Heide T, Williams MJ, Zapata L, Nichol D, Chkhaidze K, et al. Subclonal reconstruction of tumors by using machine learning and population genetics. Nat Genet. 2020 Sep;52(9):898–907.
