# Supplementary figures and images for: Inferring ongoing cancer evolution from single tumour biopsies using synthetic supervised learning
Source: PLoS Comput Biol. 2022 Apr 28;18(4):e1010007. doi: 10.1371/journal.pcbi.1010007 (PMC9049314; doi:10.1371/journal.pcbi.1010007)

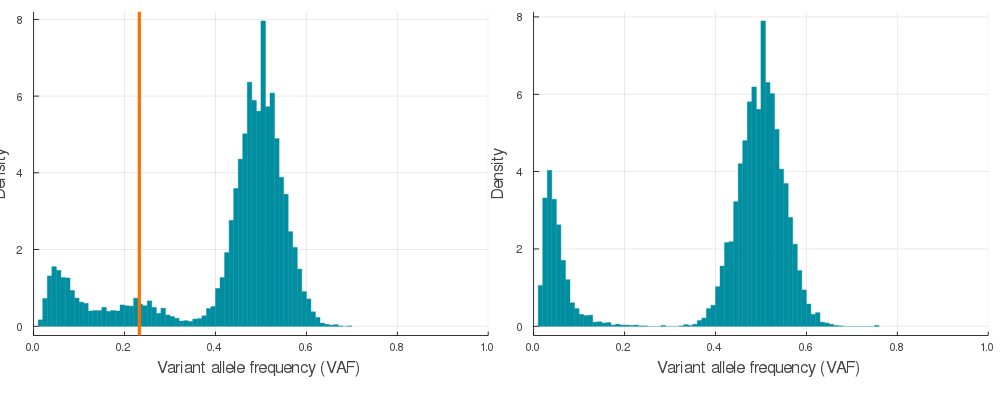

Supplement: S2 Fig — Orange lines in the positive selection plot (left) indicate the subclone frequency (cellular proportion / 2). (GIF) [file pcbi.1010007.s003.gif]

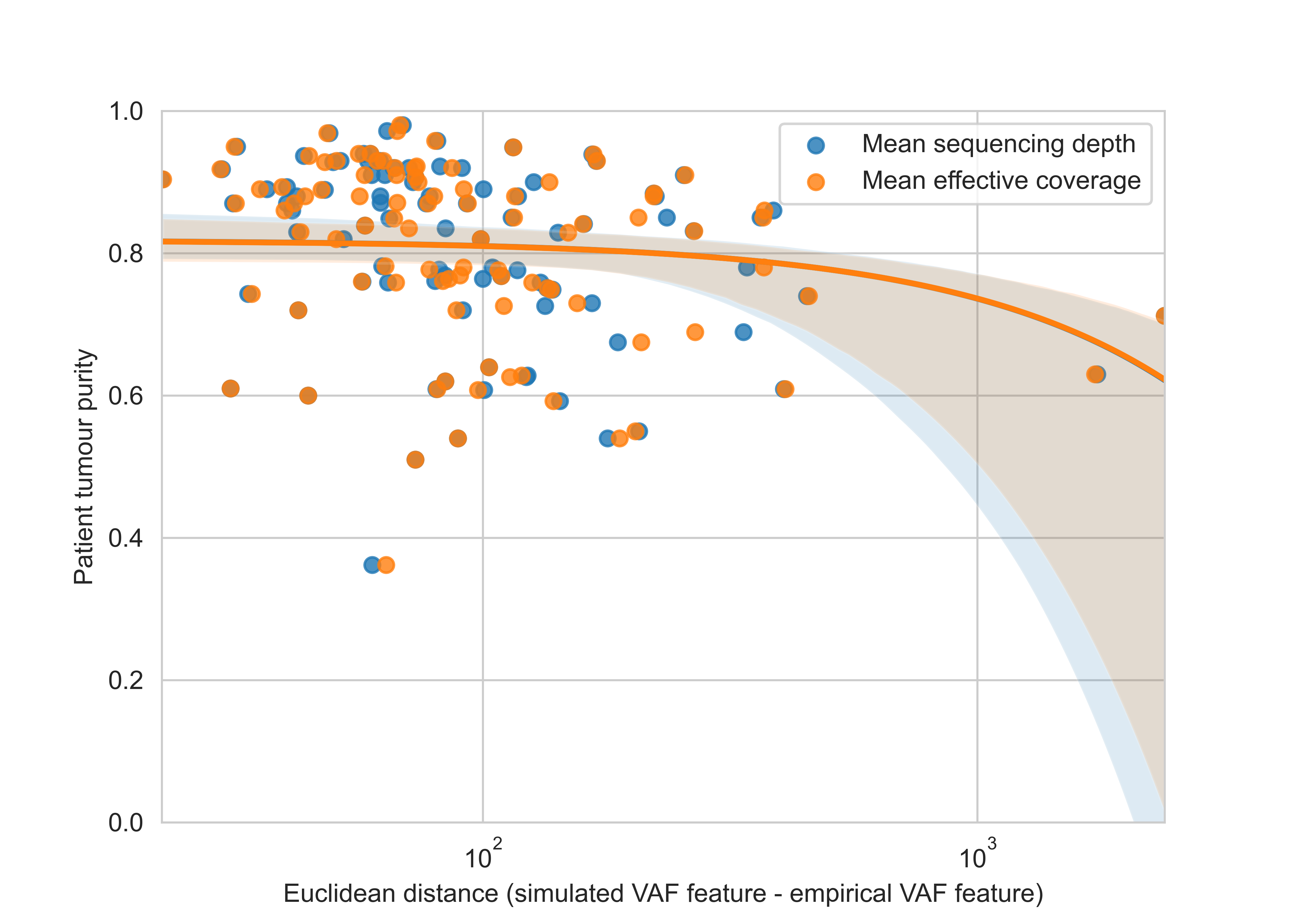

Supplement: S5 Fig — Using mean effective coverage (mean sequencing depth * purity) does not improve the fit between synthetic VAF distributions and patient tumour biopsy VAF distributions as defined by the euclidean distance of the nearest neighbour. Furthermore, samples only show modest increase in euclidean distance with decreasing purity which suggests the synthetic data generation scheme is robust to modest reductions in purity (e.g., > 0.5 analyzed in this study). (PNG) [file pcbi.1010007.s006.png]

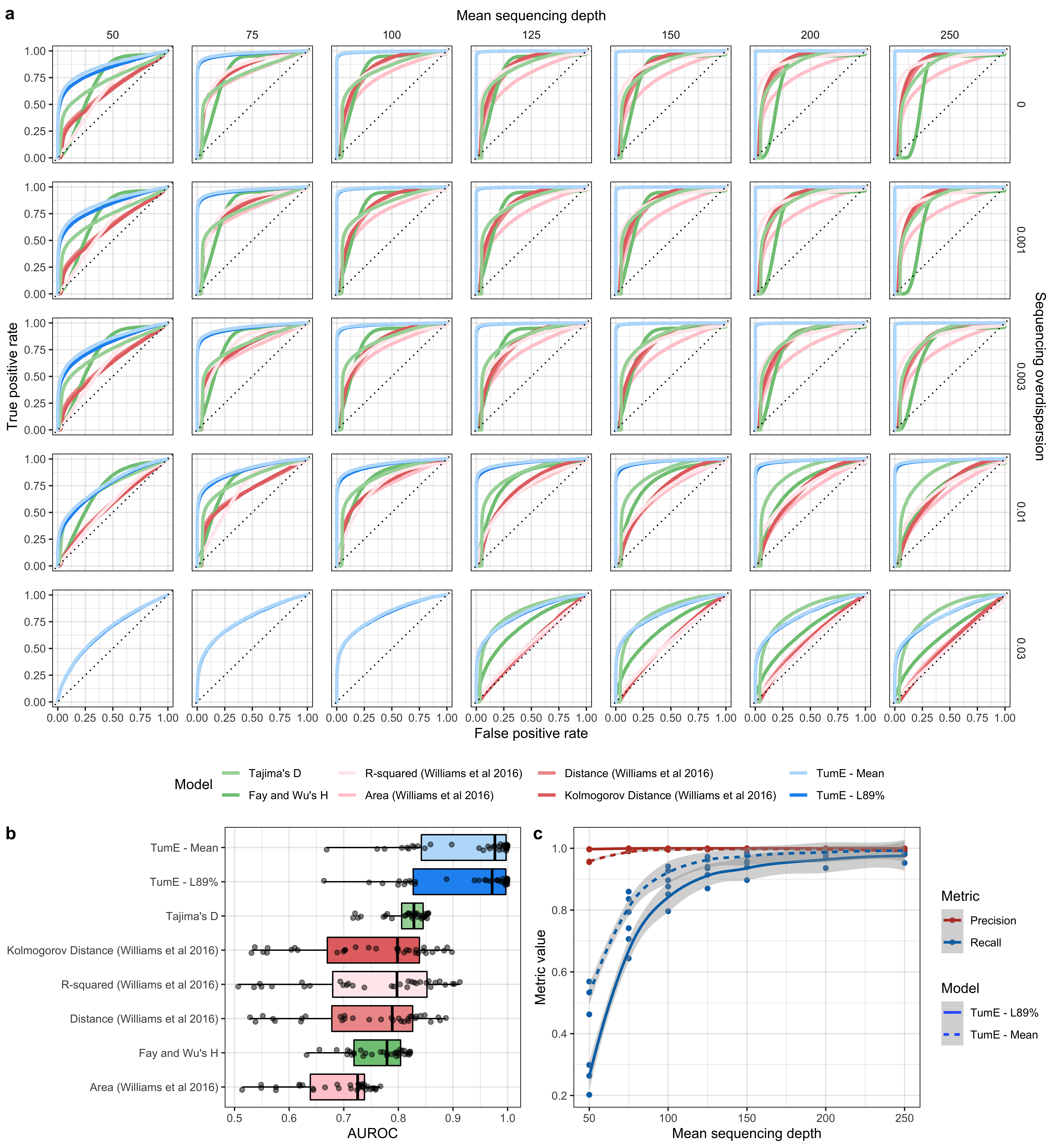

Supplement: S7 Fig — To evaluate each method, we simulated approximately 2.8 million synthetic tumours across different mean sequencing depths (50, 75, 100, 125, 150, 200, 250x) and sequencing overdispersion parameters (0, 0.001, 0.003, 0.01, 0.03). Sequencing overdispersion refers to the rho parameter of the beta distribution in a beta-binomial sequencing noise model. An overdispersion parameter of 0.01 is a rough upper limit for empirical data. The mutation rate was set to 100 mutations per cell division per genome. (a) ROC curves for each sequencing depth and dispersion combination where columns indicate sequencing dispersion (see top text) and rows indicate sequencing depth (see right text). For each non-TumE statistic, the optimal threshold/cutoff value for specifying positive selection was determined by maximizing the sensitivity and specificity in a cutpoint analysis using the cutpointr package. (b) The median AUROC across all sequencing depth and overdispersion parameters for each method. TumE—Mean refers to calling positive selection if the mean of the approximate posterior density was greater than 0.5 for P(Selection). TumE—L89% refers to calling positive selection if the lower bound of an 89% equal-tailed interval was greater than 0.5 for P(Selection). (c) The precision and recall for calling positive selection using different approximate posterior cutoffs (mean or lower bound of 89% equal-tailed interval) at increasing sequencing depth. (PNG) [file pcbi.1010007.s008.png]

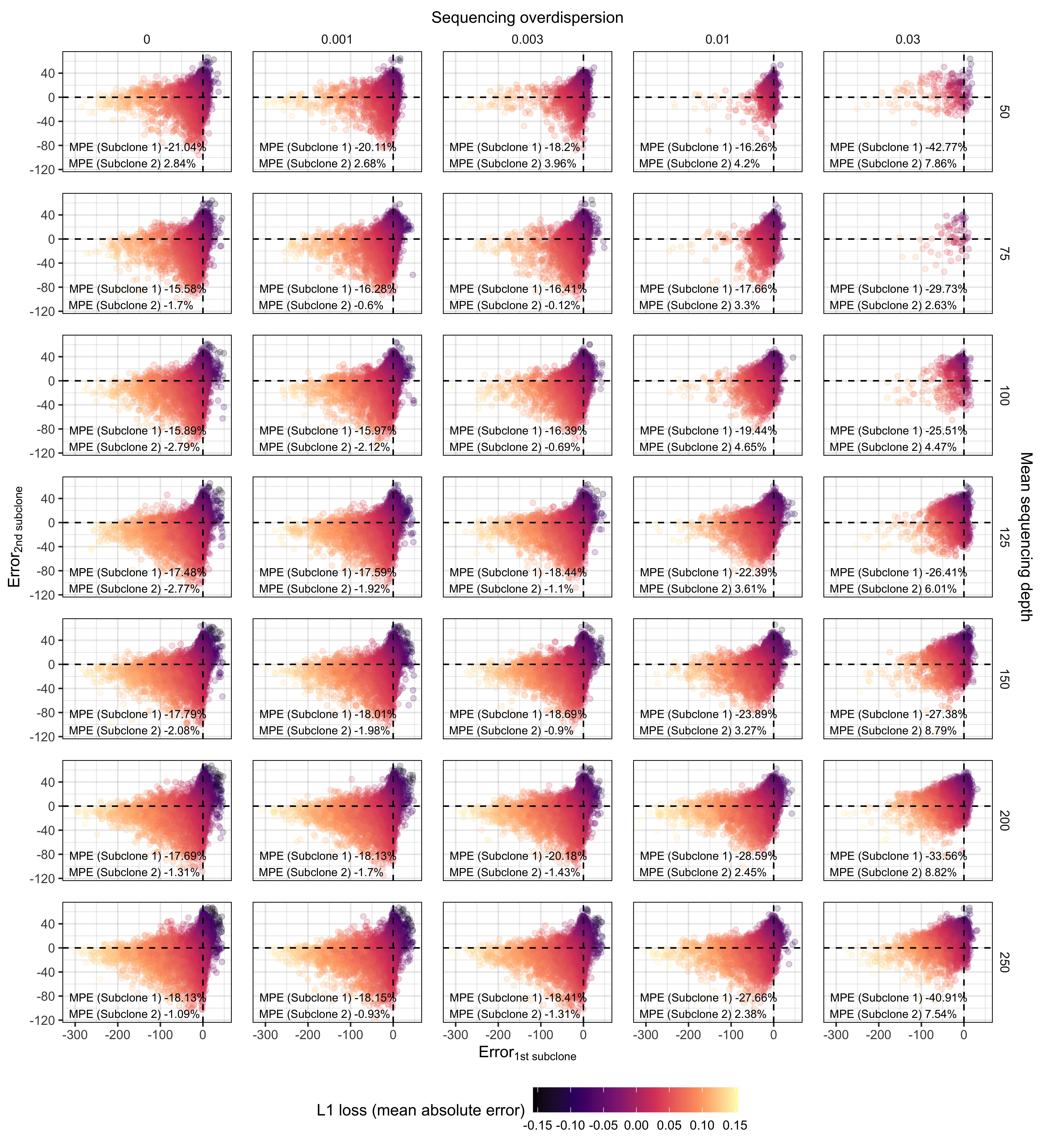

Supplement: S10 Fig — We simulated approximately 500,000 synthetic tumours with 2 subclones located between 9–41% VAF. Our simulations covered sequencing depths from (50 – 250x) and sequencing overdispersion parameters (0–0.03). Each panel shows the predicted error (true frequency—predicted frequency) for the highest frequency subclone (Subclone 1) and lowest frequency subclone (Subclone 2) in each of the 500,000 VAF distributions/synthetic tumours. The mean percentage error (MPE) for predicting Subclone 1 and 2 is provided at each sequencing depth and overdispersion combination. The color of each point is the L1 loss or mean absolute error for each sample. (PNG) [file pcbi.1010007.s011.png]

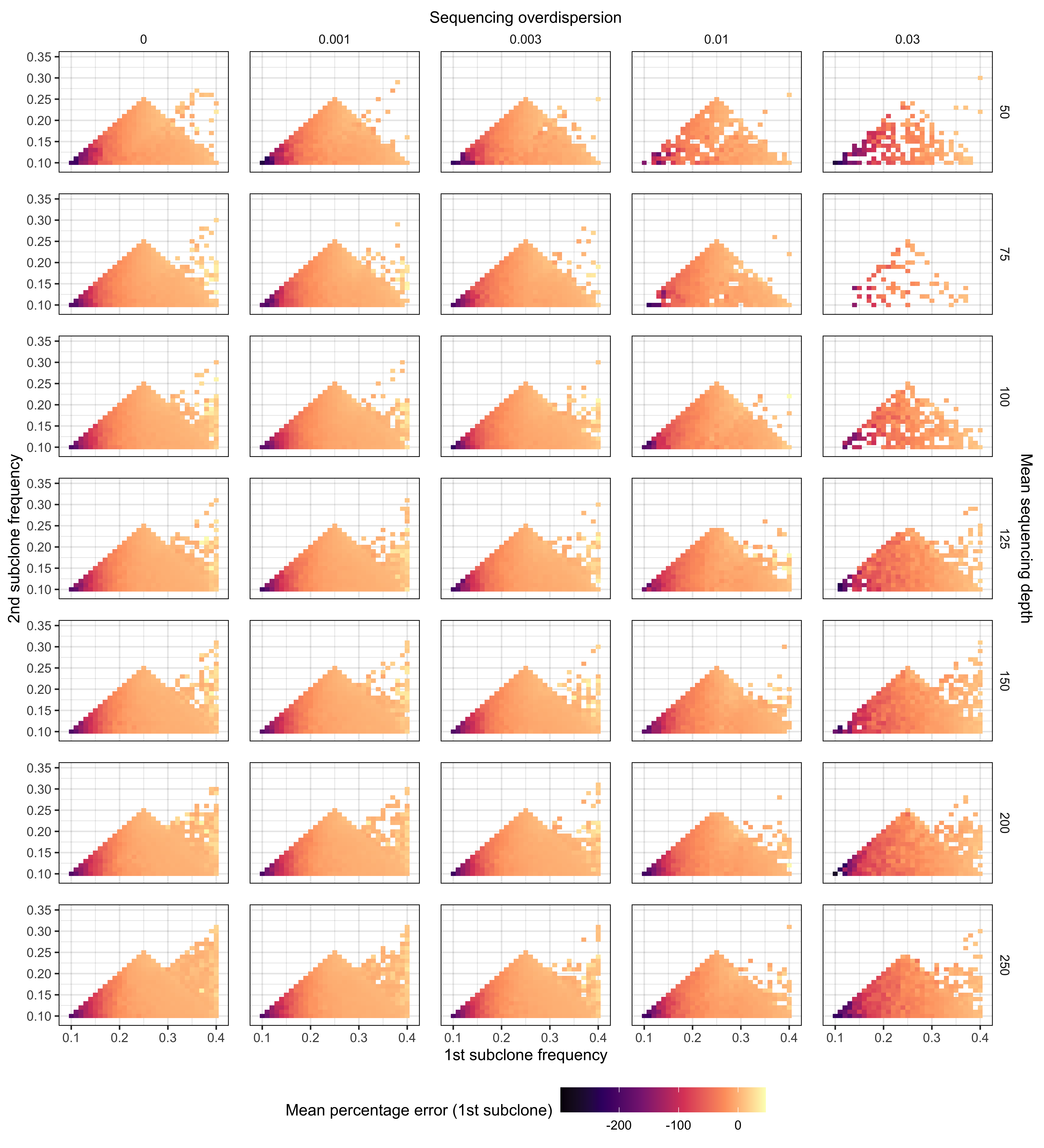

Supplement: S11 Fig — We simulated approximately 500,000 synthetic tumours with 2 subclones located between 9–41% VAF. Our simulations covered sequencing depths from (50 – 250x) and sequencing overdispersion parameters (0–0.03). For frequency intervals/bins of 0.1 VAF, the mean percentage error was computed for predicting the 1st subclone frequency. (PNG) [file pcbi.1010007.s012.png]

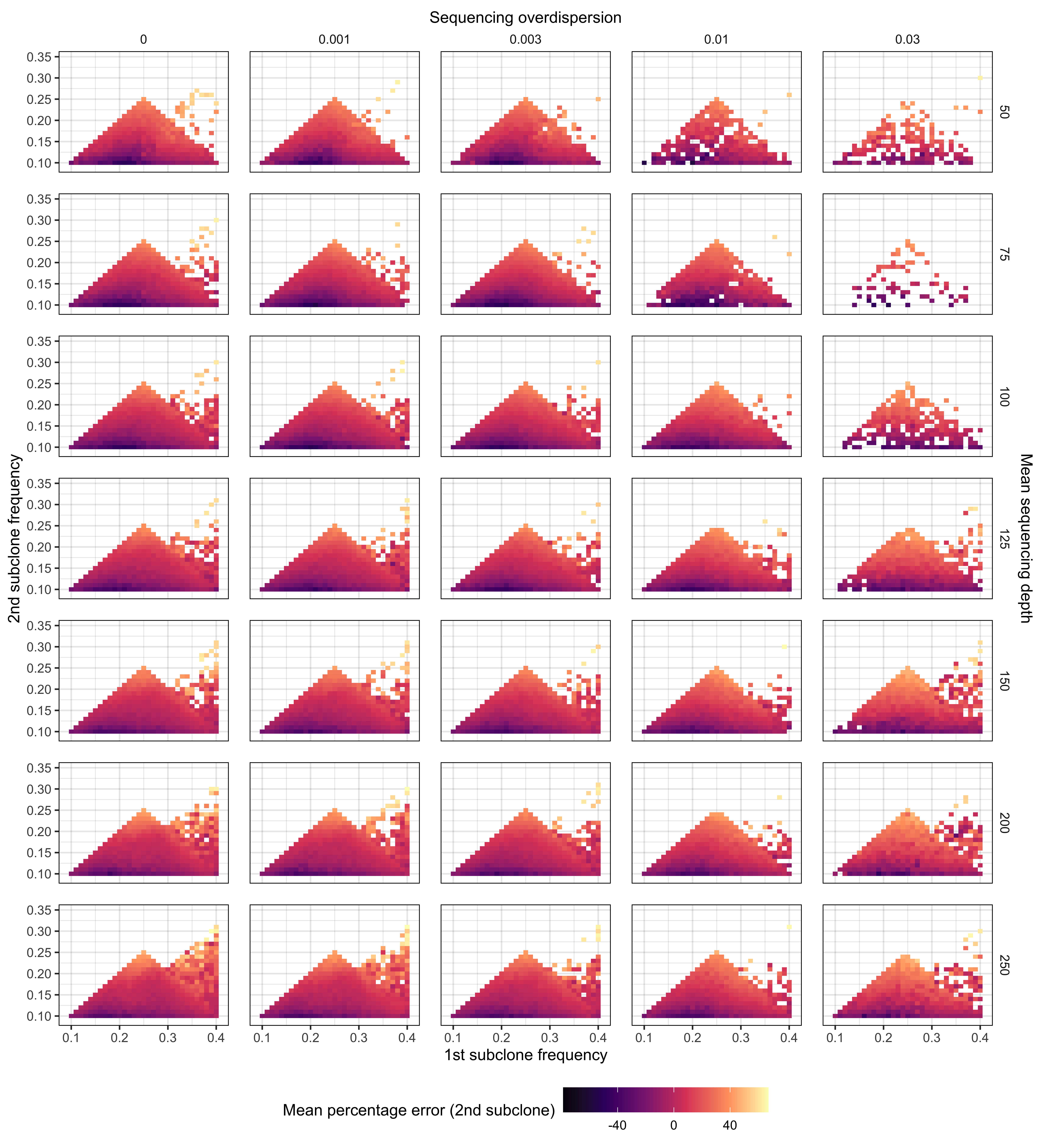

Supplement: S12 Fig — We simulated approximately 500,000 synthetic tumours with 2 subclones located between 9–41% VAF. Our simulations covered sequencing depths from (50 – 250x) and sequencing overdispersion parameters (0–0.03). For frequency intervals/bins of 0.1 VAF, the mean percentage error was computed for predicting the 2nd subclone frequency. (PNG) [file pcbi.1010007.s013.png]

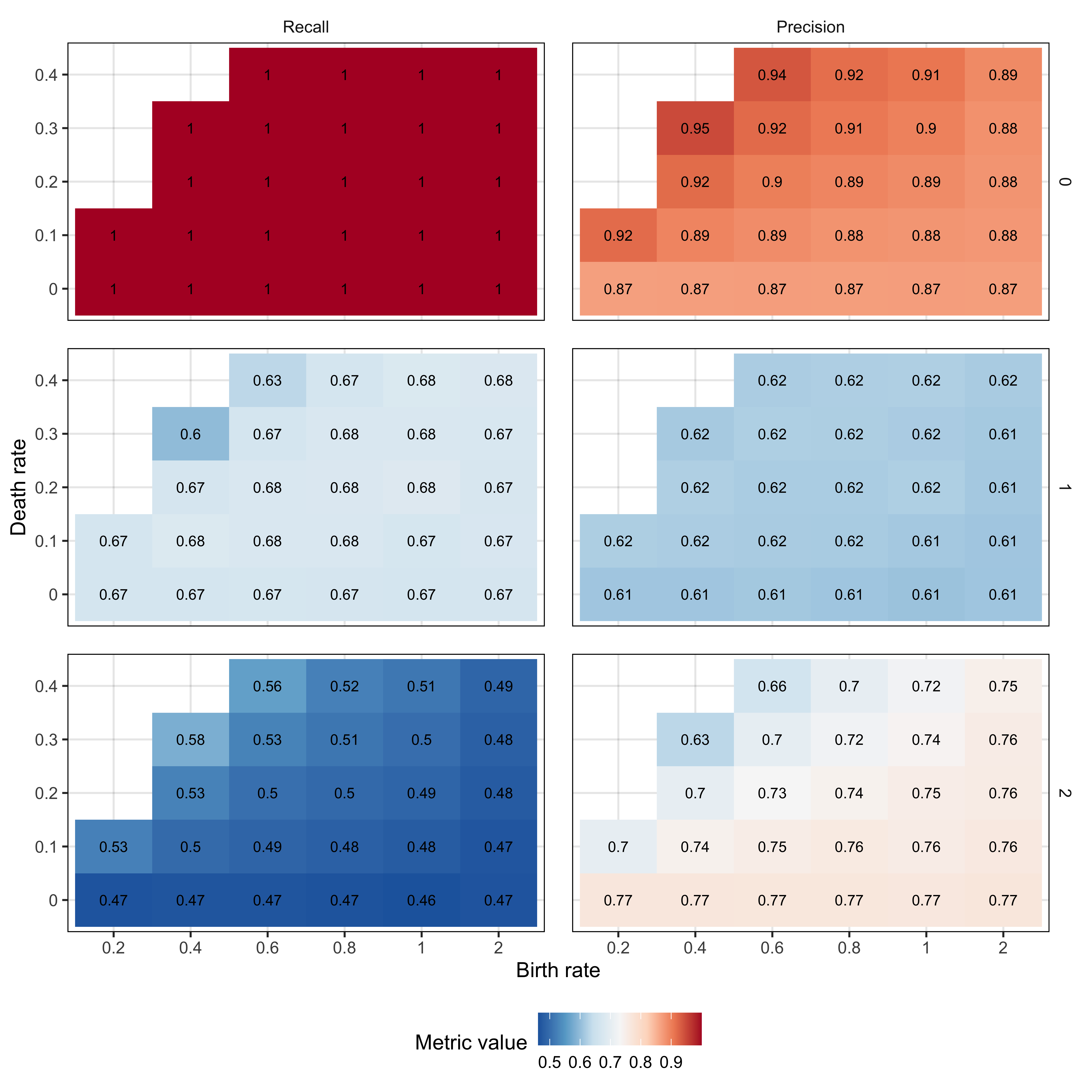

Supplement: S15 Fig — We evaluated model performance under different birth rate and death rate combinations as we built our inference models using data generated at a birth rate of log(2) and death rate of 0. We find that our performance is consistent across all birth rate and death rate combinations analyzed, although we are more likely to overestimate two subclones when only one subclone is present at low birth rate and death rate combinations (e.g. birth rate = 0.4 and death rate = 0.3). (PNG) [file pcbi.1010007.s016.png]

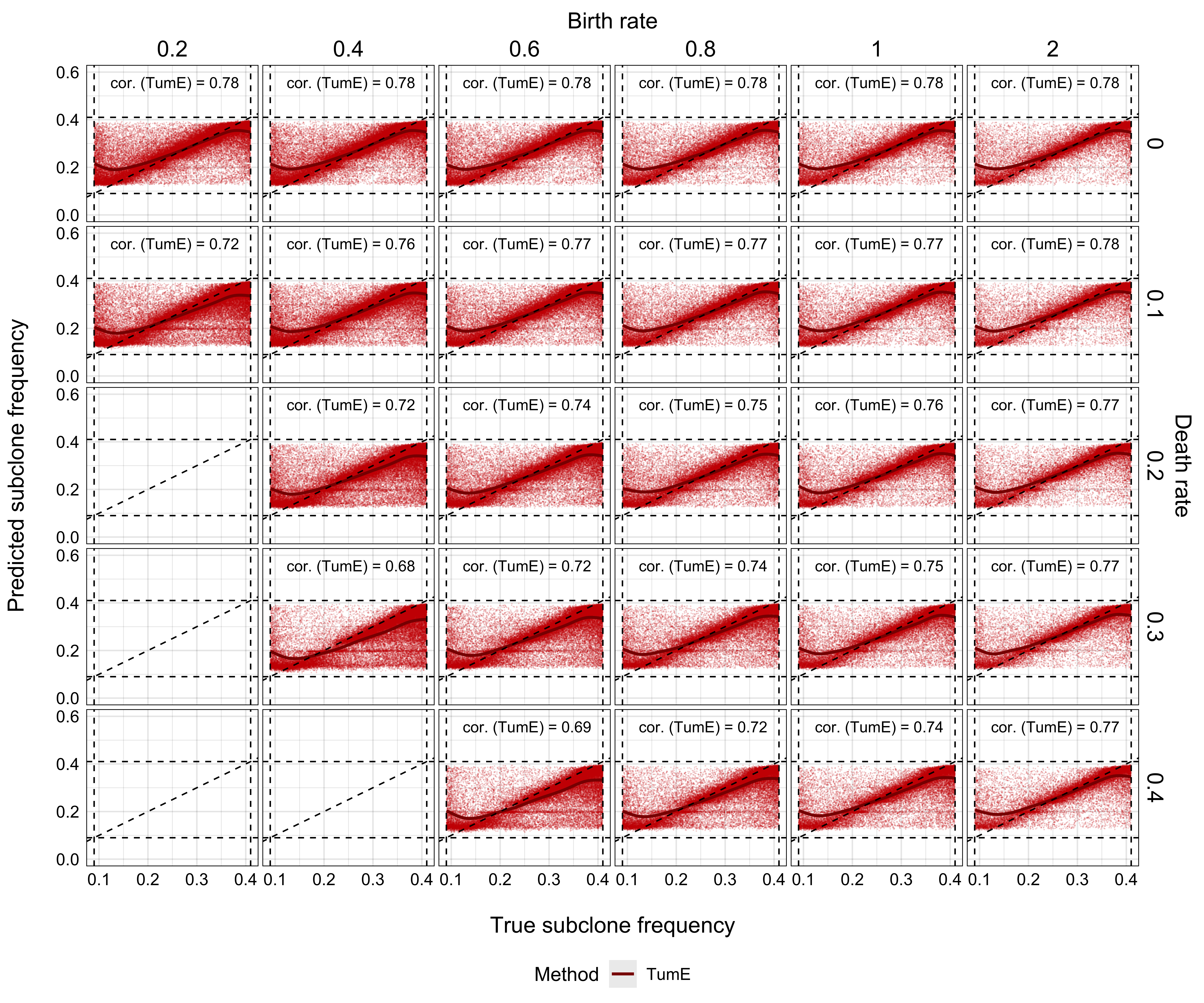

Supplement: S16 Fig — We evaluated model performance under different birth rate and death rate combinations as we built our inference models using data generated at a birth rate of log(2) and death rate of 0. We find that our performance is consistent across all birth rate and death rate combinations analyzed. Correlation between true and predicted subclone frequency provided in top right corner of each facet. (PNG) [file pcbi.1010007.s017.png]

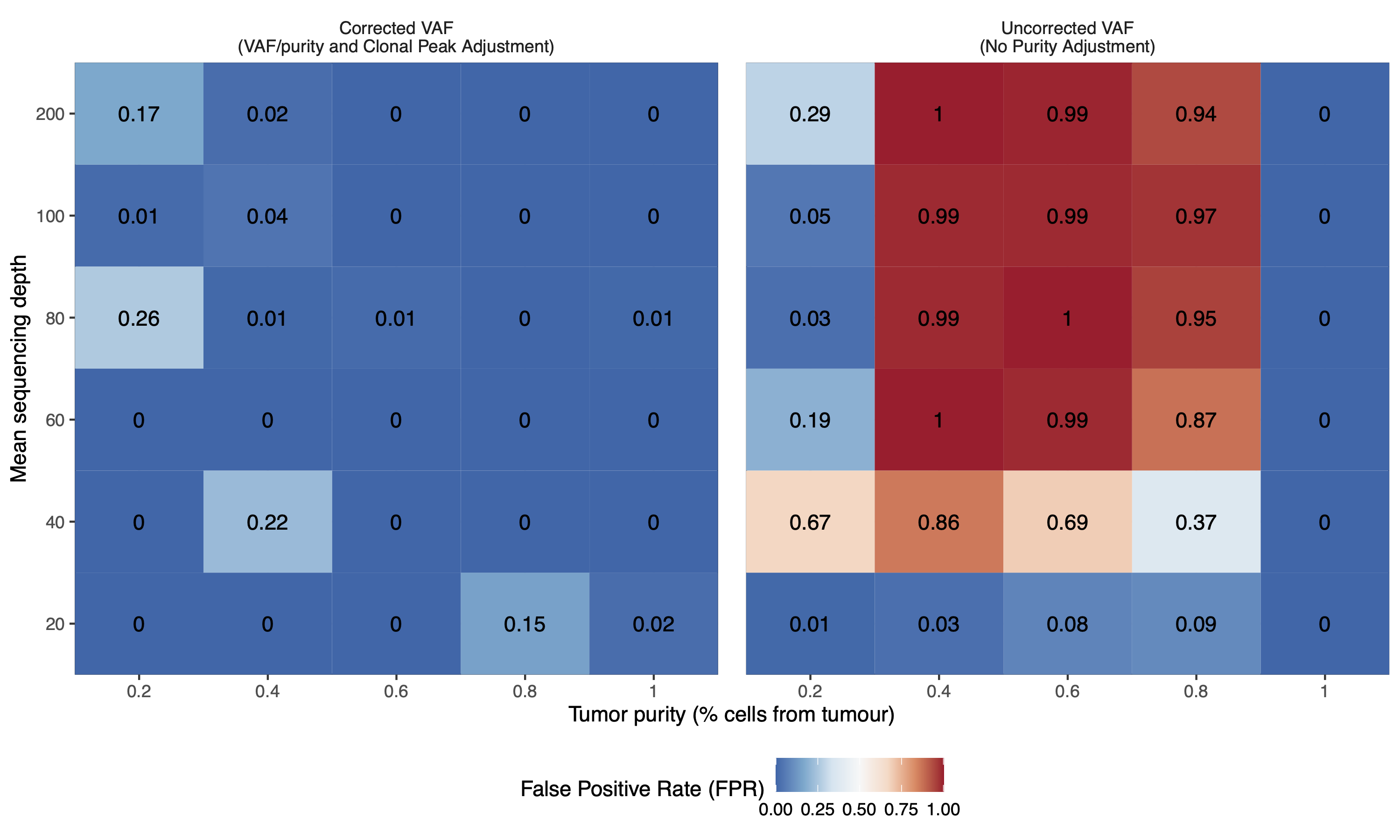

Supplement: S17 Fig — We evaluated TumE estimates in 6000 tumours with and without correcting for tumour purity. Tumour purity ranged from 0.2–1 and sequencing depth ranged from 20–200. Our analyses suggest tumour purity correction taking into account clonal peak adjustment and diploid purity adjustment (i.e. VAF/purity) is necessary to ensure accurate prediction in low purity samples. (PNG) [file pcbi.1010007.s018.png]

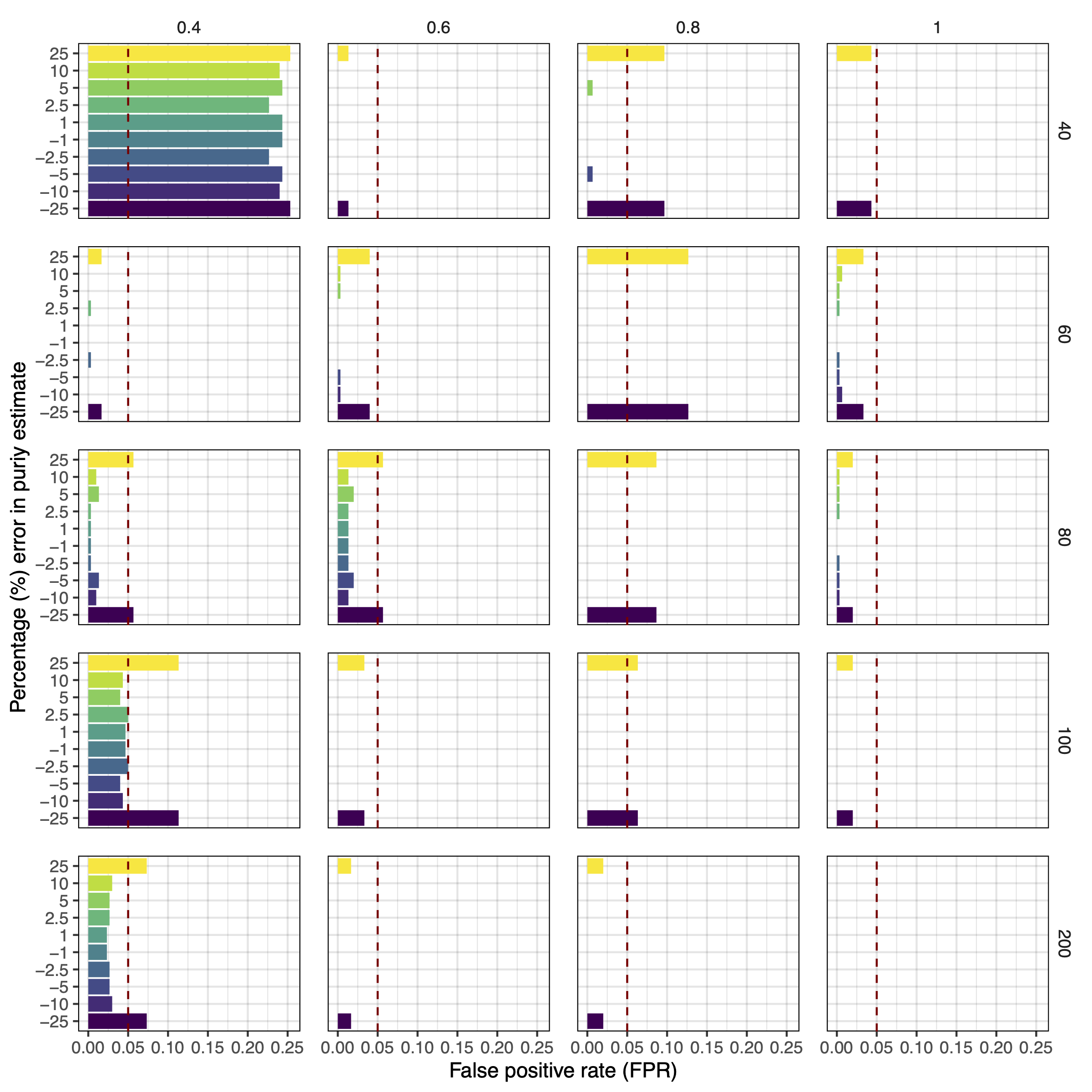

Supplement: S18 Fig — We evaluated TumE estimates in 6000 synthetic tumours following the addition of different levels of incorrect purity estimates ranging from 1 to 25% above or below the true purity. VAFs from each synthetic tumour were corrected using the peak-finding/heuristic adjustment method described in Methods. We found TumE estimates using this adjustment were robust to errors in purity estimates up to 25%, maintaining a <5% false positive rate at mean effective sequencing depths of ~40x. The top labels on each panel column is the true tumour purity. The right labels on each panel row are the mean sequencing depth for each synthetic tumour. (PNG) [file pcbi.1010007.s019.png]

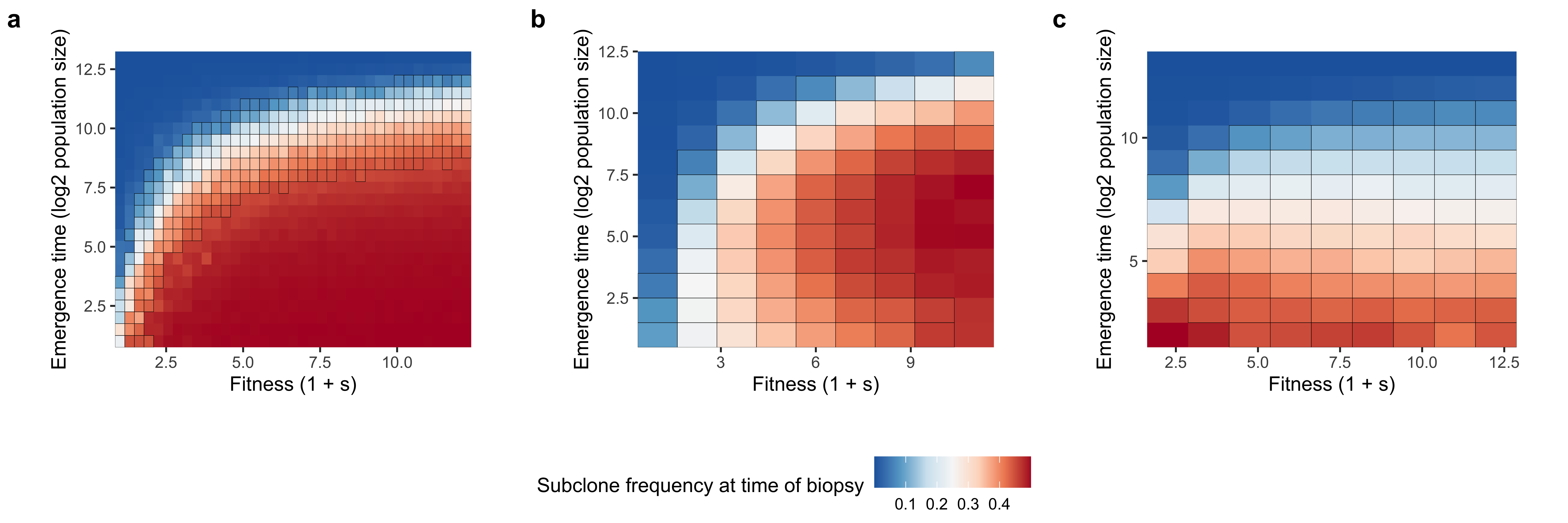

Supplement: S20 Fig — Before generating a complete synthetic dataset, we examined the subclone fitness and emergence time that led to detectable subclone frequencings using the TEMULATOR simulation framework. Synthetic tumours were initialized with 500 clonal mutations and then simulated at a birth, death, and mutation rate of 1, 0.2, and 20 to a final population size of 10,000 cells. Mean sequencing depth was set to 100x and sequencing noise was generated under a beta-binomial model. (a) Subclone frequencies at varying fitness and emergence time combinations in the 1 subclone setting. (b, c) Subclone frequencies at varying fitness and emergence time combinations in the 2 subclone setting where the latest arising subclone is (b) and the earliest arising subclone is (c). We note that we only consider the 1 subclone case when validating the transfer learning approach in this paper. (PNG) [file pcbi.1010007.s021.png]

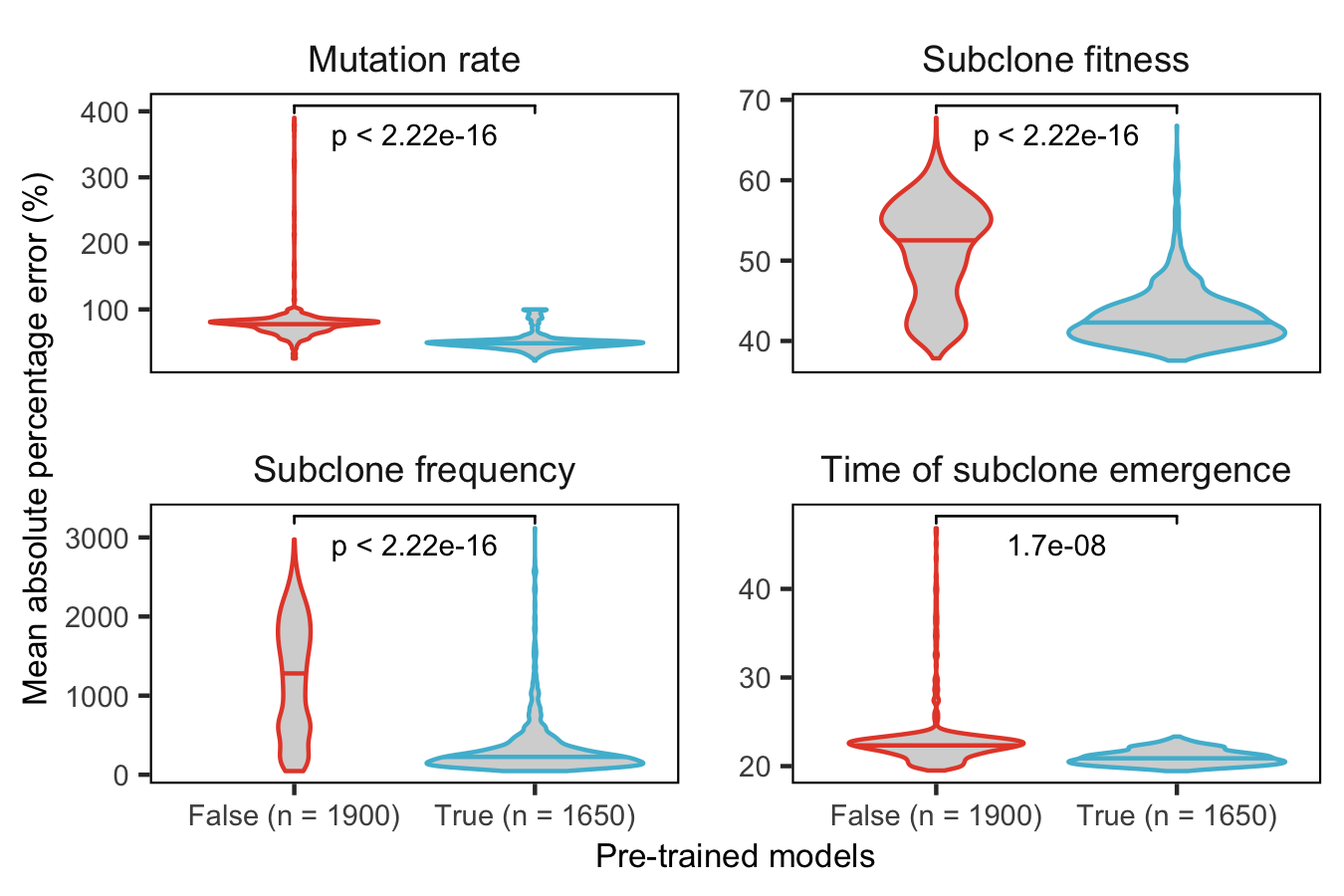

Supplement: S21 Fig — To exhibit the benefit of using pre-trained models when performing related evolutionary inference tasks, we evaluated the mean percentage error for predicting four TEMULATOR parameters/variables in a test set of 400,000 synthetic tumours. Non-pretrained models (False) and models with pre-trained convolutional layers (True) were trained on 500,000 synthetic TEMULATOR tumours for a maximum of 10 epochs. Hyperparameters (number of fully connected layers in new task specific branches and learning rate) were tuned via random search. Predictions for each model (38 non-pretrained & 33 pre-trained) were made across 50 sequencing depth and mutation rate combinations (e.g. 100 depth and 50–100 mutations per genome per division; n = 1900 & n = 1650). (PNG) [file pcbi.1010007.s022.png]

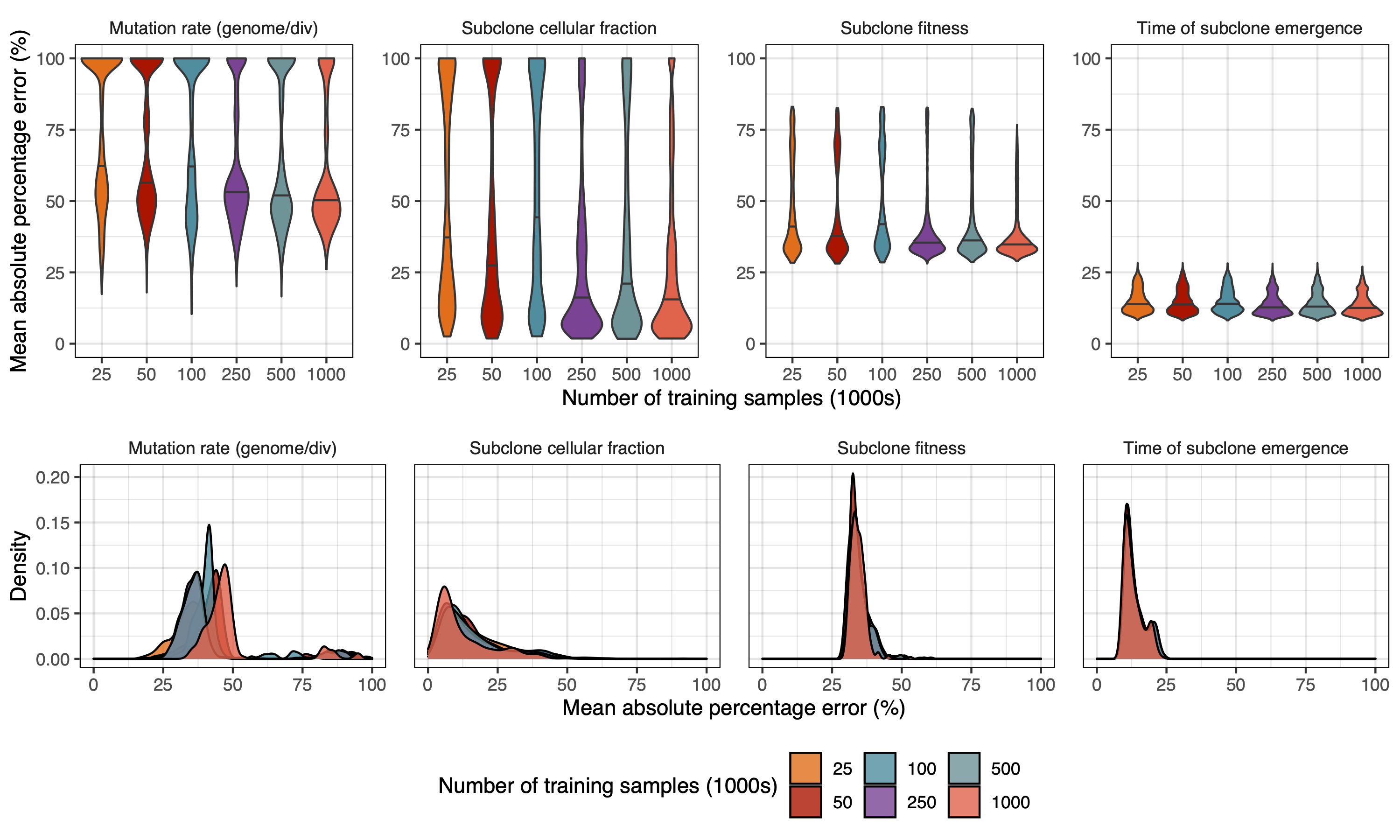

Supplement: S22 Fig — (top row) (Top row) Distribution of mean absolute percentage error (MAPE) versus training set size with 100 models generated via random hyperparameter search per training set size. Each model was evaluated in 500,000 synthetic tumours simulated at mean sequencing depths of 75–200 and mutation rates ranging from 0–500 mutations / genome / division. Only synthetic tumours containing a subclone between 20–80% subclone cellular fraction were included. (Bottom row) Evaluating MAPE in the top model (based on minimizing MAPE) from each training set size. For each model, MAPE was computed across 300 subclone cellular fraction (0.2–0.8, step 0.1), mutation rate (0–500, step 50), and sequencing depth (75, 100, 125, 150, and 200) combinations. (PNG) [file pcbi.1010007.s023.png]

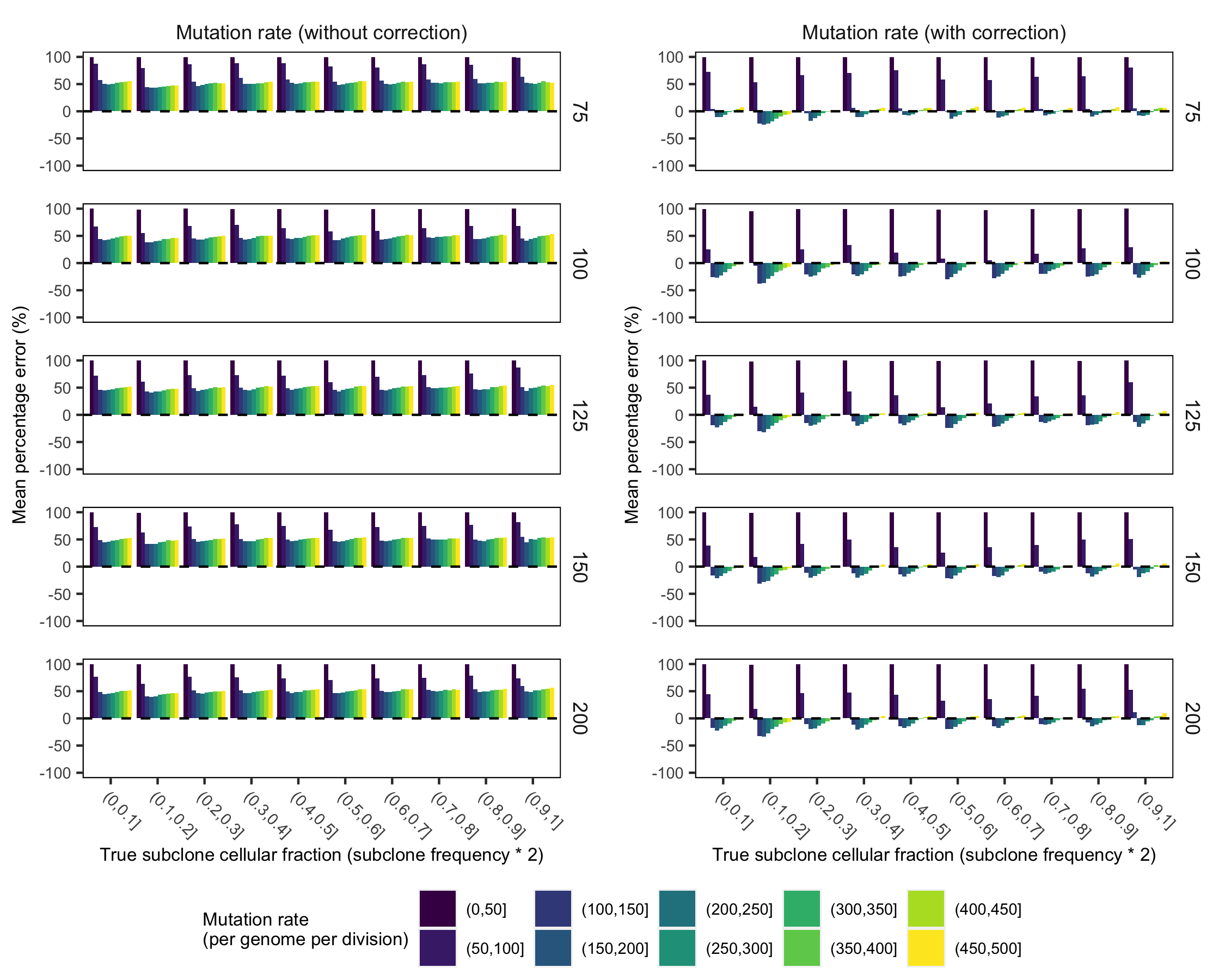

Supplement: S23 Fig — (a) We observed a modest but systematic underestimate of mutation rate following model training (as obseved in the positive mean percentage error). To correct this underestimate, we fit the outputted mutation rate estimates with a polynomial regression (degree 2) to predict the true mutation rate in a training set of 1000 synthetic tumours. (b) We then validated the correction in a holdout set of 100,000 synthetic tumours. The correction properly adjusted the majority of mutation rate estimates although lower mutation rates remained reasonable but underestimated. The labels on right axis of each plot indicate mean sequencing depth. (PNG) [file pcbi.1010007.s024.png]

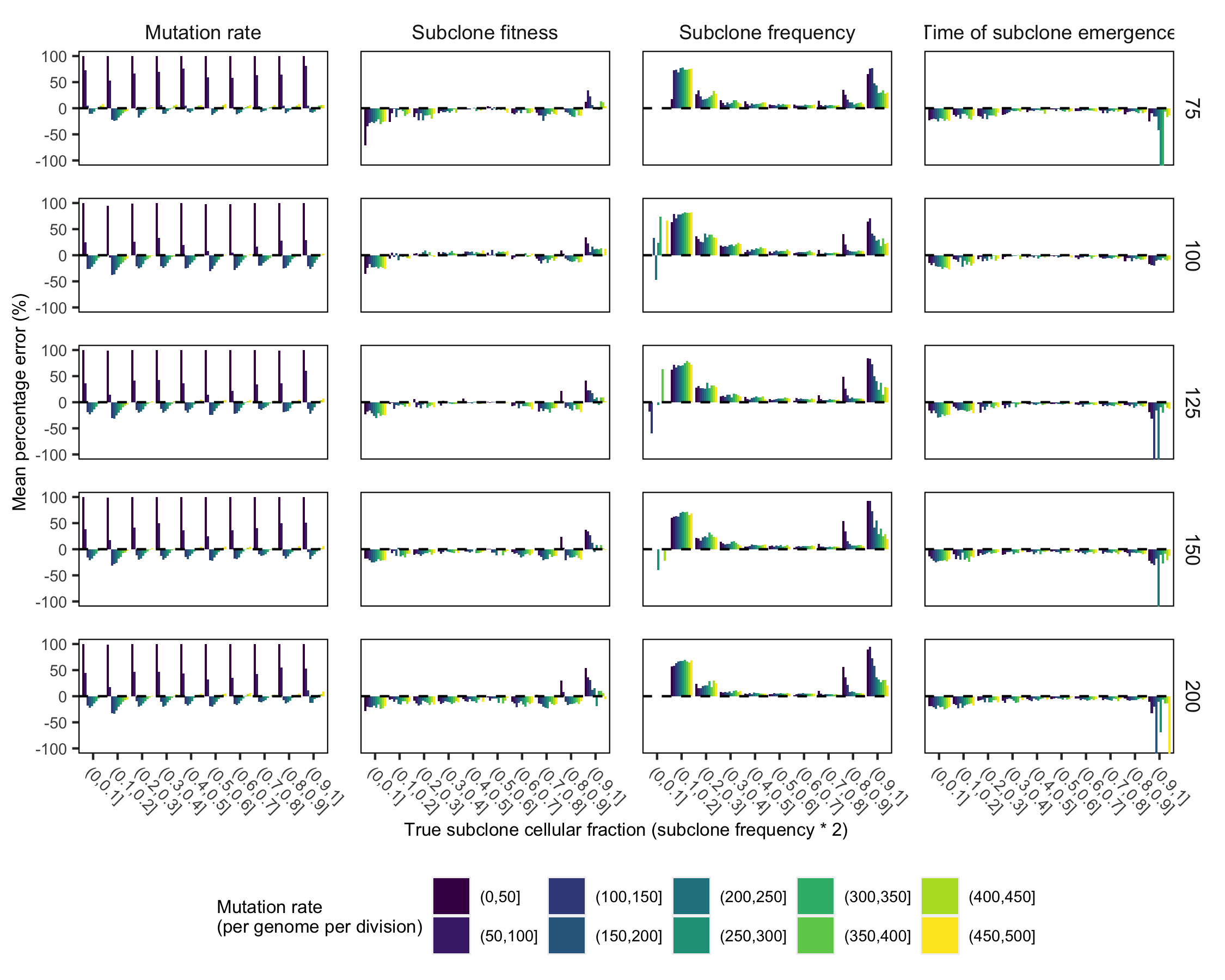

Supplement: S24 Fig — Mean percentage error for predicting mutation rate (per genome per division), subclone cellular fraction, subclone fitness (1 + s), and subclone emergence time (in tumour doublings) with top performing TumE transfer learning model. The labels on the right axis of each plot indicate mean sequencing depth. (PNG) [file pcbi.1010007.s025.png]
