# Supplementary material for: Inferring ongoing cancer evolution from single tumour biopsies using synthetic supervised learning
Source: PLoS Comput Biol. 2022 Apr 28;18(4):e1010007. doi: 10.1371/journal.pcbi.1010007 (PMC9049314; doi:10.1371/journal.pcbi.1010007)
Supplement: Attachment — Submitted filename: reply_to_reviewers.docx [file pcbi.1010007.s031.docx]

Reply to Reviewers for paper **“Inferring ongoing cancer evolution from single tumour biopsies using synthetic supervised learning”**

**Reviewer 1**

**Reviewer #1:** The authors have provided a method for generating synthetic bulk tumor sequencing data by modifying an existing algorithm. They also designed and implemented a novel deep learning model and train it on the synthetic data to capture tumor evolution, i.e. detect positive selection, identify number of subclones, and estimate subclone frequencies.

The data synthesis method and the deep learning model are well-designed and the authors show that the model can learn the patterns of the synthetic data well under different testing scenarios. They also show that the CNN embedding can be used for transfer learning. However, simulation-based machine learning models are only as good as the assumptions underlying the data synthesis process. In other words, only realistic assumptions during the data synthesis can result in trained models that are applicable to real-world data.

To that point, I believe the authors have made a fair amount of effort to show the validity of the simulated data. First, they use a well-established data simulation framework, i.e. stochastic branching, as the basis of their suggestions. Second, the authors evaluate their model on orthogonal synthetic data to showcase its generalizability. Third, it is shown in the supplementary figures 3 and 4 that some of the synthetic data are similar to real-world data from patient tumors. Fourth, the model has been able to perform well on 'gold standard' real data.

Given the computational accuracy and speed of the proposed solution and the novelty of the methods, I recommend this manuscript for publication.

**Reply**

We would like to thank the reviewer for their positive comments, and for their excellent summary of the main points/highlights of our paper.

**Reviewer 2**

**Reviewer #2:** This paper discusses the use of deep learning and transfer learning, coupled with Bayesian inference, to infer the evolutionary history parameters relevant to whole-genome or whole-exome sequencing of tumour samples. It is well-written and coherently argued, but there are several limitations that I would like to see addressed before the article can be published.

1) Although the software tool is easily downloaded and installed, the data used in the provided example (which comes from a public AML dataset) is not easily available. It would be imperative to ensure reproducibility by either providing a complete pre-processing procedure for this dataset or providing an already pre-processed version in the online supplements if that is an option.

**Reply**

All original and pre-processed samples (and scripts) were previously published in the referenced Zenodo repository (data availability statement) under /dataset/seq/. We have updated the higher-quality PCAWG samples (>60x depth) in the folder /revisions/v2/pcawg/ in the Zenodo repository. However, we would still like to thank the reviewer for their comment as we noticed a bash script was missing to re-generate all the results and figures with one command-line call. This single bash script has been added to the updated (version 2) Zenodo repository.

2) This paper discusses transfer learning, but only in one direction (from other simulators to TEMULATOR), which begs the question of whether the transfer could be effective in the other direction (say, from TEMULATOR to other simulators). Also, although the amount of data used for retraining is relatively small (less than a million samples vs. tens of millions of samples), it is large in absolute terms, and it would be interesting to see a discussion of how the accuracy of the inference changes with the size of the retraining sample.

**Reply**

We thank the reviewer for the great suggestion. We have analyzed the relationship between training set size and mean absolute percentage error for predicting mutation rate, subclone cellular fraction, subclone fitness, and time of subclone emergence using TEMULATOR and found that although  500,000 samples appeared to reach asymptotic predictive performance, as few as 25,000 synthetic samples can generate a model with comparable predictive performance assuming the hyperparameter search space is large enough (e.g. >100 models).

As a result, the following additions to paper are listed below.

- We have added a new Supplementary Figure 22 showing how the average model performance across training set size changes. This figure can be easily accessed in the online supplementary we have provided at <https://tomouellette.gitlab.io/ouellette_awadalla_2021/supplementary/4.1_transfer_learning.html>.
- We have added an additional commentary in the Results Line 370 - 373 section indicating the findings on how training set size alters performance of transfer learned models.

3) It was not clear to me why a paper focused on the evolutionary histories of tumours does not include a discussion of – or even reference to – any of the literature using phylogenetic methods (such as “SPhyR: Tumor Phylogeny Estimation from Single-Cell Sequencing Data under Loss and Error” and related one). I would like to either see a direct comparison to those methods or a complete explanation of why such a comparison is not appropriate, at least.

**Reply**

We thank the reviewer for their insightful comment. We also believe that phylogenetic methods, such as SPhyR and other single cell phylogenetic approaches, provide another modality for understanding the evolutionary history of a tumour. However, we would like to highlight that our approach is complementary, rather than competitive, with a phylogenetic approach.

- Firstly, we are working at the level of subclonal deconvolution. This means that although we ‘segment’ the subpopulations within the tumour, we do not make a claim on the phylogeny going back in time (although we could, however this may be trivial given we are largely working with 0 - 2 subclones subject to selection).
- Second, our population-based methods were also designed to incorporate inference of selection or neutrality when making estimates of the number of subclones in bulk samples; as far as we are aware such inferences are not developed within the phylogenetic based reconstructions of tumors. For example, a phylogenetic inference finds the most likely tree given the data, whereas a population-based approach can be thought of as assigning the most likely evolutionary mode over all possible ‘trees’ that could describe the data. With that said, our output of cluster assignments, for example, could be used in multi-region biopsy methods to reconstruct tumour phylogenies/histories using bulk-sequencing data.
- Third, we note that in this study we focused on making evolutionary/subclonal estimates using single-nucleotide variants (SNVs) in bulk-sequenced tumours rather than single cells. We chose bulk-sequenced data due to (i) bulk-sequencing data from tumours still being the most widely available and cost-effective for researchers and (ii) due to the current state of single cell SNV calling – namely - limited data, high costs for sufficient number of cells for evolutionary analysis, and potential issues with errors and sample quality. Nonetheless, SNV calling at the single cell level is a rapidly evolving area and we are excited to see how single cell SNV calling (beyond population-level consensus calling) expands into the future.

**Additions to paper related to our reply**

- We have added the following statement on Page 16 Line 416-419 in the discussion to discriminate how a population-based approach distinguishes itself from phylogenetic approaches. "Lastly, in contrast to phylogenetic approaches that use mutations to reconstruct or explain the possible history of divergent clonal events in a tumour biopsy, population-genetic based methods, such as TumE, discriminate detectable subpopulations subject based on explicit evolutionary models such as positive selection or neutral evolution using the VAF distribution”.
- We have added 2 references related to phylogenetics in the above passage, namely a review of tumour phylogenetics in ref 40 Schwartz & Schaffer (2017) and a single cell method in ref 41 El-Kebir (2018).

**Reviewer 3**

**Reviewer #3:** This paper presents TumE, a Deep Learning approach to tumour subclonal deconvolution. The main idea is to train a deep classifier to predict K - the number of tumour subclones - from synthetic tumour evolution datasets generated through a branching process model. The data-generating algorithms are taken from previous works by Williams et al., and comparison is carried out against 2 mixture-based methods (Sciclone and MOBSTER), with only one (MOBSTER) that accounts for neutral tails in tumour evolution.

**I praise the following aspects of this work:**

- reproducibility: almost all code is present and quite well documented. While this might seem obvious to a computational scientist, this is not the standard attitude in the field.
- Deep Learning: while we are not huge DL practitioners, we found the idea of training over simulations the most interesting aspect of this paper.

**Reply**

We thank the reviewer for their kind comments on our work. We aimed to document our code for ease of reproducibility and insist that, if on further inspection, anything seems unclear when using our code to open an issue in an associated github repository.

The DL model - once trained - is obviously faster than mixture models, even all models are very fast, and TumE seems to be more precise in some scenarios. Clearly TumE has limitations in terms of interpretability and other features that are instead clear in mixture models (mutation assignments, and flexibility to scale to any subclonal architecture).

**Reply**

We thank the reviewer for their thoughts here. We would first like to list a few general points (based on the paragraph above) before addressing the remaining comments below:

- We also agree there are trade-offs between model choice, but would like to highlight that theoretically, a synthetic supervised learning approach can be scaled to arbitrary subclonal architecture. We chose two subclones because this appears to be the upper bound when using VAF information generated from single-site tumour biopsies sequenced to depths common in data available from pan-cancer cohorts.
- Further, as you note below we also do technically enable mutation assignments after the means of subclonal peaks have been identified. In this case, although you rightfully state it uses the same clustering distributions as mixture model approaches, we would argue our approach is potentially more flexible than any single mixture model as subclonal mutation assignments can now be assigned trivially with *any distribution* of choice. This is because we assign subclone frequencies *before* clustering, whereas in standard mixture model approaches subclone frequencies are determined *after* clustering. In addition, given the speed of our method for identifying the location of subclones, we also highlighted in the Discussion (Line 326) that our model could potentially be used synergistically with mixture models i.e. for example “... providing fast, evolutionary-informed peak initializations for mixture model based methods.”.

Overall, one aspect of the TumE model that should be considered is the adherence of the training set to real data, especially when the tools are applied to real datasets (in this sense some extra work has to be carried out).

**To improve the work we discuss major points below.**

Major points (computational aspects for the training set):

- Overall, the method provides an interesting approach to the problem of reconstructing the subclonal structure of a single tumor sample, taking it from an unsupervised to a supervised learning setting and allowing for greater functional complexity than models that rely on mixtures. However, the network was trained on a set, which although huge, is composed only of simulated data. Ultimately, the quality of the tool will depend on how well these simulations are able to generalize to the real data. Despite the effort in generating the simulations we believe that some naive assumptions are made regarding how really "complex" is patient genomics data (heavily harbouring somatic alterations, with noise from upstream bioinformatics analysis, noise from specific sequencing technologies etc.). We therefore think it is important to consider also:
  - The effect of miscalled CNVs segments in the tumour profile. The effect of such segments is to re-shape the VAF distribution, generating false VAF peaks (confounders). It would be nice if TumE could predict K - number of clones - but also propose "alternative" explanations such as "miscalled CNVs" (here, non-diploid segments called as heterozygous diploud). In this ways, a tumour would be "polyclonal" unless miscalled CNVs are a possible alternative explanation of data peaks (the user might afterwards screen off one model versus the other with complementary analyses). This will be crucial to make sense of TumE's predictions on real data (see below). Can such error probability be included in the learning process? Can TumE decide whether data has strong-enough signals to support one model over the other in an undisputable way?

**Reply**

We thank the reviewer for this suggestion. Indeed, miscalled CNAs would impact all methods of population or phylogenetic reconstructions of tumor histories including our ability to correctly identify subclonal populations. However, this problem is one that must be addressed by all methods. A reasonable approach to this problem is to use high-quality consensus copy number calls to limit CNV errors leading to spurious peaks in the subclone frequency. We use the same samples from MOBSTER and follow the same stringency for only using the high-quality consensus calls as defined in PCAWG.

However, assuming some degree of error still does occur, we note that a subclone must be of reasonable size to actually reject the neutral evolution hypothesis. This can clearly be observed in Supplementary Figure 6. At sequencing depths of ~70x, subclones need to carry upwards of 20% of total mutations to actually lead to the lower bound on P(Selection) to be > 0.5 (We use the lower bound of the 89% interval to call selection). This means that a CNA error has to lead to an increase in ~20%  of subclonal mutations for there to be an impact, which seems unlikely as most errors will be rare.  So even if some error (CNA)  does occur, the error has to be reasonably large to lead to a spurious subclonal call. In any case, the above speaks to errors that could impact VAFs and suggests that a reasonable filter will remove most of these and is not problematic. A fully interactive version of Supplementary 6 where the user can filter through different trained models can be found here: <https://tomouellette.gitlab.io/ouellette_awadalla_2021/supplementary/2.1_mc_dropout_estimates.html>.

**Additions related to reply**

- For clarify, we have added two comments to discussion and methods to clarify the emphasis on high-quality copy number calls
  - Discussion, Page 16 - 17, Line 432 - 436 “...copy number variants miscalled as diploid can lead to overestimation of subclonality by introducing spurious mutations in the subclonal frequency range. However, in this study, we focused on using high-quality consensus copy number calls extensively validated in pan-cancer cohorts and utilized in previous work^16,34,35^
  - Methods, Page 24, Line 720 - 724, “ … similar to ref^16,34,35^, we only used high-quality diploid consensus calls (star 3) from the original PCAWG study to avoid potential biases from miscalled segments appearing as subclones in the VAF distribution. Samples with fewer than 100 mutations following removal of low-quality regions were not included in this study.”

After the MOBSTER paper, the quest of whether a dataset harbours resolution for reliable subclonal deconvolution seems important. For instance, one conclusion of that paper is that PCAWG data are too-low resolution to trust most deconvolutions:

- "Our analysis suggests that, for most PCAWG cases, the data resolution was too low to conduct robust subclonal reconstruction. [...] Standard analyses of these data therefore risk systematically mistaking neutral tails for subclonal clusters, thus inflating the complexity of the inferred subclonal architectures and producing incorrect phylogenetic trees.".
- I interpreted that as a cautionary message - garbage in, garbage out - which I think it is reasonable because, at low-coverage/purity, certain noise sources (callers?) are difificult to control. Ouellette and Awadalla should consider if they can reliably learn to regress low-resolution data assuming - as they do now - almost a noise-free model (see other points below), or at least a model where the noise contribution is very limited to be considered "realistic".

**Reply**

We also believe the MOBSTER paper was an important step forward in the cancer evolution space, especially for highlighting the importance of neutral evolutionary dynamics (preventing the overestimation of subclones) and data quality. Given the findings from the MOBSTER paper, we took care to implement multiple quality control steps in *simulated* and *real* samples to ensure data quality and proper specification of simulated data with respect to real patient tumour sequencing data. We note our approach to this potential confounding problem in our next reply below — we believe there are two complementary but potentially different strategies for addressing errors derived from miscalled purity, sequencing depth, etc.

- The effect of miscalled purity. While the authors shift randomly the clonal per between 45% and 50%, we believe a more detailed approach is needed (changes in purity also shift sub clonal clusters and tails). In particular, the estimation of sample purity happens together with CNV inference - i.e., the two quantities are linked. For this reason, miscalling purity often implies miscalling ploidy, and therefore miscalling clonal heterozygous diploid segments (allele-specific CNVs). The effect of these chained errors is not considered in the current problem formulation, and the consequent learning process is working in too idealistic scenarios. The authors should either try to augment their model and create more realistic datasets to train TumE, or at least have a more critical undertaking of these aspects.

**Reply**

We thank the reviewer for this comment. Miscalled purity is definitely an issue that can lead to incorrect inference if a parametric approach that relies on properly positioned clonal clusters, like our neural network based method, is used without correcting the observed VAFs. We were aware of this issue at the beginning of our study and we hope we can clarify to the reviewer how we addressed the potential misclassification of positive selection and poor data quality associated with impurity (i.e. VAF overdispersion and mismatch between true VAF and observed VAF due to impurity):

Approaches to address data quality w.r.t to purity in *simulations*

- Firstly, in relation to VAF overdispersion, we note that impurity and low sequencing depth are essentially overlapping problems – namely that both low purity or low depth will lead to a smaller amount of reads overlapping a given SNV, and the VAF distribution in general will be more overdispersed. Therefore, simulated tumours sequenced under lower depths *implicitly* captures overdispersion due to lower purity. We model sequencing noise in this study using a standard approach (Methods 470 - 480, see also Williams et al. or MOBSTER Caravagna et al papers): binomially distributed total read depth and binomial or beta-binomial alternate read counts. Therefore, in practice, lower purity leads to a lower ‘effective sequencing depth (purity * sequencing depth). In this study, we only accept samples with an effective sequencing depth >60x, and state that samples with greater than ~>70x effective sequencing depth are likely required for accurate estimates (Methods Line 726 - 729). This observation aligns with the MOBSTER paper, which we further note in Results, Line 262 - 264. As our simulated data implicitly captures overdispersion in the VAF distribution associated with lower purity, we further describe how adjustments in real data (which is present in our methods section) correct the mismatch between observed VAF in an impure sample and the expected VAF if only tumour cells were present.

Approaches to address data quality w.r.t to purity in *real data*

- Our major approach to addressing issues with purity was at the level of real patient sequencing data i.e. correcting real data to match the theoretical diploid state at 100% purity (similar to Williams et al. 2018).
- Firstly, given genome-wide linkage as the basis to study diploid regions only (selection on any genomic alteration will pull *all* linked mutations of any ploidy to higher frequency), correcting VAFs for impurity is straight forward ⇒ e.g. VAF_corrected = VAF_observed / purity.
- However, we note, as does the reviewer, that purity estimates can be wrong (see Methods @ Line 619 - 640). Fortunately, the VAF distribution provides evidence for incorrect purity estimates. In this regard, a simple heuristic involves checking if the heterozygous clonal peak (which should be at 50% VAF since these fixed/clonal heterozygous mutations *must* be in 50% of the tumour cells) is properly aligned to 50% VAF. If purity estimates, and other associated second-order effects involved in its computation, were incorrect then the adjustment of the VAF distribution will not lead to a clonal cluster correctly aligned at 50% VAF. We address this issue using a custom heuristic that locates the clonal peak and re-adjusts the VAF of *each* SNV “..by multiplying by 0.5 divided by the mean of the fit”. (Methods @ Line 738-754).

Lastly, the random shift of the clonal cluster to 45-50% is only performed in simulated tumours generated under a neutral evolutionary process. In this study neutral evolution is our *null* model. By randomly shifting the clonal cluster of *only* neutral synthetic tumours (elaborated in Methods Line 527 - 532,  we force our trained models to be less confident in estimating positive selection if the clonal cluster remains incorrect even after purity correction and heuristic adjustment as noted above.

**Additions to paper related to our reply**

- Analyzed the  impact of error in tumour purity estimates on TumE inference using 6000 synthetic tumours
  - Supplementary Figures 17 & 18 showing the robustness of our heuristic purity and VAF distribution correction; shows that TumE maintains a false positive rate less than 5%, and in most cases less than 1%, (with high precision) for positive selection even when purity estimates are upwards of 25% incorrect
  - Figures can be easily viewed in the online supplementary found here <https://tomouellette.gitlab.io/ouellette_awadalla_2021/supplementary/2.5_purity.html>
  - Extended comment with respect to this analysis in Results Line 228 - 238.
- We have added the function used for VAF distribution adjustment to the python TumE package *TumE.utils.correct_vaf* for users to adjust/correct samples with potential purity misestimates (Previously this was only available in the Zenodo repository and we originally coded it in R for use in our sample filtering pipeline).

- While randomly trimming the tail partially reproduces problems of noisy mutations at low frequency, in reality what happens is more an undersampling process, where we do see some mutations at low frequency, just way fewer than expected. Also, there is an intrinsic noise effect that is due to the particular caller adopted (certain callers adopt different calling thresholds, noise models etc.), which is also exacerbated in the case of consensus pipelines like in PCAWG, where multiple callers are adopted (what is the consensus noise model!?). Randomly chopping the tail is not necessarily neither the best nor the correct model - these aspects should be clarified. As a matter of the experience of this reviewer in working with noisy low-frequency data, whereas this shhold be perfectly power-law shaped (theoretically), it often shows exrta peaks after the effect of any caller is added up. This effect often resembles a sort of non-linear noise model because of heuristics adopted by callers (i.e., retain mutations with at least 3 supporting reads, etc.). The authors should therefore provide more evidence about the goodness and appropriateness of their random-truncating tail approximation.

**Reply**

The reviewer also brings up a fantastic point about the impact of the intrinsic noise effects of different variant callers and consensus callers. However, we believe our current approach, although a potential simplification of the various underlying sources of noise, effectively addresses this problem for the task of detecting positive selection.

- Firstly, it is important to emphasize that our synthetic generation process i.e. random trimming of tails and shift of clonal cluster *was only performed in simulated tumours generated under a neutral evolutionary process*. This means any degeneration of the ‘neutral tail’ would lead to our model calling neutral evolution i.e. our null model. Assuming parsimony, this feature is important as without a somewhat present neutral tail it may be hard to determine if a peak is a truly subclonal or part of the degenerating tail unless the subclone is reasonably large (carries many mutations).
- Secondly, we note that our synthetic data generation process is in actuality an undersampling process and not just an abrupt trimming of the data as suggested by this comment. This is because we perform trimming *prior* to injecting binomial or beta-binomial sequencing noise, thus adding sequencing noise post-trimming effectively undersamples/disperses the tail. This can be observed in pseudo-algorithms provided in Supplementary and starting at Line 554 in our simulation source code located at <https://github.com/tomouellette/CanEvolve.jl/blob/main/src/simulation.jl>.
- Lastly, we note that the detectable subclonal range is ~10-40% VAF. This means, in practice, we ignore peaks within the neutral tail (~<10% VAF)  as, the reviewer rightly noted, there are generally multiple technical explanations for variability in this portion of the VAF distribution. As such, variation in consensus calling or number of minimum reads of 1,2,3,4.. have no impact on prediction at mean sequencing depths considered in this study.
- In general, we believe our nearest neighbour search between simulated and real data in Supplementary Figures 3 - 5 is a great qualitative example of the proper specification of our simulated data w.r.t to patient data. With observation, it is clear that downsampled or degenerated ‘tails’ are well-specified using the synthetic data generation scheme. A fully interactive figure that allows for iterating through all patient samples and nearest simulated neighbours can be found here: <https://tomouellette.gitlab.io/ouellette_awadalla_2021/supplementary/1.2_simulation_check.html>

**Additions to paper related to our reply**

- We have added a comment in the methods to improve clarity on the order in which trimming and sequencing noise (which generates a downsampled neutral tail) occurs in the ‘Synthetic data generation’. Methods  Line 528 - 535 “.....by adding sequencing overdispersion following trimming, we naturally capture the observed downsampling of degenerate neutral tails in empirical data.”

- Mutation assignments: a foundamental feature of mixture models is the explicit modelling of a latent responsibility per mutation. TumE regresses K over the VAF, producing a multinomial estimate of the number of subclones, and just afterwards takes care of assigning mutations to clusters. The process of clustering assignements is performed by using the correct model, but still it is completely decoupled from the regression. The authors should comment more on this and the potential pitfalls - in the end, the authors claim that they can learn complex non-linear VAF/data relations, but finally cannot perform clustering assignments with anything than a standard approach, therefore losing potential advantages of the non-linear mapping. In this sense, mixtures are ahead of TumE. Also, given the presence of ground truth data where assignments are known, quantitative measures of performance on miscalled assignments must be provided. For this purpose, mutual information and related measures should be used to provide a quantitative measure of how TumE assignements are off, also relative to other models adopted (SciClone and MOBSTER).

**Reply**

The goal of our paper was to predict important evolutionary parameters in bulk tumours whereas mutation assignment is more related to dealing with the additional noise effect from sequencing that leads to, in general, symmetric overdispersion in clonal and subclonal peaks (not considering neutral tails). Therefore, we believe because sequencing noise is in essence a post-hoc effect, clustering mutations using binomial variance, or potentially gaussian variance, combined with our subclone frequency estimates is a sufficient and reasonable way to implement this additional feature. As noted above, we detect subclones frequencies *before* clustering, therefore clustering assignments have no impact on our frequency estimates, whereas with mixture models, clustering and subclone frequency estimation must be simultaneously solved. In regard to mutation assignments, it’s not entirely obvious how the non-linearity provided by neural networks when predicting subclone frequency would aid in overcoming the uniform randomness in mutation assignment when subclone clusters overlap. cont’d in bullet points>

- As noted in the PCAWG heterogeneity group paper in Cell (<https://www.sciencedirect.com/science/article/pii/S0092867421002944>)(see their Methods section ‘Performance on simulated data’), mutual information is not necessarily a useful criteria in quantifying performance of subclonal detection methods – generally because uniform random assignment of mutations occurs if clusters (such as a subclone peak and clonal peak) are overlapping.  Because of this, in the Cell paper, they opt not to use mutual information or related metrics like rand index to quantify this task – this is also because, in general, if you can make an accurate estimate on the true subclone frequency, which is definitely more difficult, then clustering is generally straightforward because the generative process for sequencing noise is known.
- In this study, we follow the general outline as the Cell paper in assessing performance for predicting the most biological important information for this problem, namely predicting the mode of evolution, number of subclones, and subclone frequency. We believe we chose proper metrics to evaluate performance of these tasks, namely using AUROC for positive selection vs neutral evolution, precision/recall for number of subclones, and correlation and mean percentage error for subclone frequency.
- This comparison against MOBSTER in simulated samples can be found in Supplementary 7 - 12 which is easily accessible in the online supplementary found here <https://tomouellette.gitlab.io/ouellette_awadalla_2021/supplementary/2.2_model_performance.html>.

Major points (computational aspects for the training set):

- One aspect is not completely clear to us. As far as we understand, the TEMULATOR population genetics model by Heide et al is the same of the Julia model by Williams et al. The simulation model by the authors is also a re-implementation [*] of the model by Williams et al, with some modification that augment generality but do not describe completely different population genetics processes. Therefore, we do not understand if learning parameters of TEMULATOR after training on simulations à la Williams should be, or not, a great surprise. We would be more surprised if parameters of completely different models were learned by transfer learning - probably after suitable transformations. We are however certain about one thing: learning decoupled growth and death rates is very interesting - probably the most interesting thing, since all other parameters are analytical from VAF fits according to Williams et al. In general, a more thorough discussion about these simularities among the generative models would help this reviewer to appreciate that part of the paper.
- [*] The main differences claimed regard the possibility of randomly sampling certain parameters, which are constant in the Williams/Heide models to speed up simulation. While this includes random effects that add learning overhead - i.e. the stochasticity of the probability of a mutation to be driver - it is not clear how different are the 3 proposed models (Heide, Williams, Ouellette).

**Reply**

We agree that this is a reasonable observation, given that both the Williams et al. and Heide et al. simulations are gillespie-like SSA simulators. However, we hope the reviewer can appreciate that the main goal of the transfer learning section was to show that learning the most basic information about evolutionary dynamics, such the mode of evolution (selection vs neutral) and number of subclones, encodes sufficient knowledge to infer more complex information, such as subclone fitness/mutation rate/timing subclone emergence, in a more efficient manner. In addition, it appears the only parameter computed analytically In Williams et al. is subclone fitness, which is dependent on parameters inferred via ABC. Given subclone frequency, subclone time, and mutation rate are all inferred via ABC in Williams et al., it’s not entirely clear that we should be able to infer additional parameters such as fitness, timing, and mutation rate with significantly fewer data points (1000s vs millions; we do note however that MOBSTER does provide estimates on time using a modified formula based on the # mutations in a given subclone). We believe we are the first to show this type of transfer learning approach in an evolutionary context, and think our exposition of  transfer learning for improving future models provides a fruitful baseline for further pursuits by us or others. For example as the reviewer suggests, in deciphering decoupled growth and death rates in tumour populations

**Additions related to reply**

- We have softened the language around simulator differences to avoid overstating any results for the reader at Results Line 344 “...built under a *similar* but modified set of assumptions…

Major points (real data applications, partially overlapping with the points regarding trainig data):

- In the analysis of 85 PCAWG samples the authors claim to detect positive selection in ~38% of cases, compared to ~96% in the original PCAWG analysis and ~3% in Caravagna et al. There is clearly a strong point of controvercy across all analyses, but I see 2 problems with these summary statistics:
  - Samples set: it should be clarified if the pool of samples upon which these analyses are carried out is the same (clearly is not), otherwise comparing these summary statistics is pointless or requires, at least, some caution. The overall PCAWG paper analyses the full set of samples (~3000), the paper by Caravagna et al. focuses on a subset of samples with high-purity and high-coverage in order to determine samples that have high-enough signal quality to determine the site frequency spectrum of the tumour's neutral tail, plus subclones. This paper selected 85 cases - "spanning 8 different cancer types, retrieved from the pan-cancer analysis of whole genomes (PCAWG)" - where the rationale for sample inclusion seem to be explained in Supplementary Figure 18 (with constraints on sample purity and coverage), but this does not match up with the one in Caravagna et al, for instance. With this inconsistencies we cannot be confident about the meaning of the proposed comparisons, and therefore the reliability of conclusions. This aspect has to be clarified, and any statment about "subclonal selection" in real data needs to be weighted by careful evidence.

**Reply**

We thank the reviewer for this comment. We were working with a limited subset of the PCAWG data at the time of study inception but have now accessed the complete set of whitelisted samples. Using this larger dataset we have increased the stringency of our filtering for high-quality samples for better comparison between methods. We used the newly accessible complete PCAWG set to filter for samples (78 in total) that had at least a 60x mean effective coverage (mean sequencing depth * purity). We believe the effective coverage threshold better captures both the purity and sequencing depth requirements for samples suitable for evolutionary inference, and is based on initial data quality not a post-hoc metric like presence or absence of a neutral tail.

**Additions related to reply**

- Analysis of the updated 78 PCAWG samples with TumE, MOBSTER, and sciClone
  - We have analyzed the updated 78 PCAWG samples with TumE, MOBSTER, and sciClone using recommended settings for each method
  - We provide all fits for TumE, MOBSTER, and sciClone in Supplementary Tables S3 to S5.
  - We provide commentary on this analysis in Results Line 286 - 309
  - We provide additional commentary on (i) how sample selection across different studies may confound rates of positive selection detection and (ii) how filtering samples based on data quality e.g sequencing depth is a better strategy than using post-hoc metrics like a neutral tail in Results 311 - 328.
  - We have added another figure (Figure 4) associated with this analysis
- In relation to the above analysis, we believe we have better contextualized the potential variability in detected rates of ongoing selection from Results Line 300 - 320 and note that the actually rate in the Cell PCAWG paper was 95.1% = ~95%  and have corrected this error

- Data suitability: here it comes an important limitation of having trained TuME on "perfect" data. The perfect training set of TumE is ideal compared to real data where somatic calling is likely imperfect. In practice, the authors seem to have taken for granted copy number estimates from PCAWG (and indeed adopted just a purity adjustment). Errors in CNA calling will create artificial bumps in the data distribution, which will "confound" VAF-based inference tasks, giving the illusion of subclones. Similarly, subclonal CNAs - as defined in PCAWG - might also affect the VAF disitribution (are they to be removed in your analysis?). With reference to Supplementary Figure S18, the authors should show, for each one of the 85 cases, the result of TumE analysis on the 85 samples, subclonal clustering and potential neutral mutations assignments. Heuristics should also be adopted to assess wethere the input data for TumE is consistent with the assumptions of the method itself. Otherwise, it is difficult to assess the quality of the inference of the proposed method on real data. Once this analysis has been properly carried out, how many subclonal drivers (at least, simple mutations) can the author assign to the identified 40% of subclonal expansions? Also, can the authors present the analysis with alternative models (MOBSTER, Sciclone) for these samples? Are they consistent and, if not, where do they differ?

**Reply**

As discussed above, we have articulated the real issues with CNA associated errors and our strategy for errors in CNV calls in response above.

In regard to “input data for TumE is consistent with assumptions of the method itself”, we may be misunderstanding the resolution at which this question is being asked. However, we evaluated the specification of our synthetic VAF distributions with respect to patient VAF distributions using a nearest neighbour search. A fully interactive analysis that allows iteration through PCAWG samples and others is available here <https://tomouellette.gitlab.io/ouellette_awadalla_2021/supplementary/1.2_simulation_check.html>.

**Additions related to reply**

- We have added all TumE clustering fits and subclone frequency assignments for neutral samples and samples with 1 subclone to Supplementary Figure 19. This can be found in the online supplementary at <https://tomouellette.gitlab.io/ouellette_awadalla_2021/supplementary/3.1_wgs_overview.html>. In case image resolution is an issue, we have also appended a high resolution PDF to the PNG version of this figure in the standard supplement. As suggested by the reviewer diploid SNVs from each sample were annotated with known driver mutations and corresponding functional impact (synonymous and intronic mutations were not annotated). A total of 26% of samples carried a nonsynonymous, indel, or splicing mutation in a known driver gene. Further description of this analysis is provided at Line 278 - 284 of the discussion.
- In regard to “Also, can the authors present the analysis with alternative models (MOBSTER, Sciclone) for these samples? Are they consistent and, if not, where do they differ?”, we described the comparisons in the reply above. The commentary within the paper on how the methods differ can be found in Results between Line 286 - 309.

Minor points:

- Difference in performance: TumE is far more fast than all the methods. The authors indeed report that (Supplementary Figure S9)
  - "[..] we note that the mixture model approach took approximately 24 hours using 500 cores to analyze >1 million synthetic tumours. Conversely, the trained neural network took a similar amount of time while using only one core."
  - However, we feel that all methods are quite fast, also considering that they provide more information than TumE concerning clustering assignments etc. We do actually feel that all tested methods are adequately fast, providing results in about <<10 minutes with real data samples. Also, we would like to note that not even at ICGC... there are millions of tumours to run. Historically, I guess one of the problems in the field was that, a few years ago, and certainly at the time of PCAWG, the speed of Monte Carlo samplers like those in DPClust, PyClone (before its Pyclone-VI extension), etc. were a real bottlneck. Nowadays, once that more powerful variational models (like in Sciclone) or MLE estiamtes (like in MOBSTER) , I am not sure "speed" is still a bottleneck; the authors could comment on this.

**Reply**

Speed is definitely a very debatable metric and the reviewer poses a great question, on how fast is enough? In most cases within a bioinformatics context, 10 minutes is definitely considered ‘fast’. But if the long-term goal of computational research is to liberate computation for every user, then having tools that can be used with near instantaneous speed and minimal computational overhead is essential. As well, MOBSTER and SciClone being non-parameteric means they scale poorly with increasing number of mutations — while the parametric approach of TumE enables essentially constant time complexity with increasing mutations. In this case, we were very conservative and generous with timing speed of mixture models in this study as the orthogonal dataset from the MOBSTER paper only included samples with ~800 - 1350 mutations. In reality, real WGS tumours carrying >2000 - 10000 mutations are relatively common and MOBSTER/SciClone can easily take over >>10-20 minutes (which they do in many samples). In general, existing methods can take hours in a sample of ~10-30 tumours with the added side effect of high CPU/memory usage whereas we provide real-time feedback with limited computational cost in seconds.

- Performance of alternative models: it seems that MOBSTER has been run fitting with more configurations then other methods. In particular, if we understand right it has been running selecting bewtween models with and without tails on simulated data, but in the end, you do not distinguish if the "tail" cluster is fit to a tail or not. Given the number of K-values tested (1,2,3 as of limits in TumE), isn't this testing twice the fits with this method? If yes, shouldn't you at least divide by 2 its computational time?

**Reply**

We do indeed have to use the tail fits to distinguish between 1 or 2 subclone fits with MOBSTER (e.g. 3 beta components without a tail are considered 2 subclones). This is because 1 subclone is the most parsimonious explanation if (a) a neutral tail, clonal beta component, and additional beta component are fit or (b) if 3 beta components fit we can assume, via parsimony, that the 1st cluster is the clonal, 2nd is subclonal, and 3rd is the tail. The new Figure 4 highlights this, as in many cases a neutral tail won’t fit the VAF distribution as a power-law component requires a ‘hard’ cutoff in the low frequency range. In these cases a 3rd beta component is fit to the tail instead. See Methods Line 672-679 in “Model performance of synthetic tumour sequencing datasets” for a complete description of the number of subclone assignment for MOBSTER.

- It is generally true the DL can provide very complex non-linear representation of the input data, it would be interesting to analyse some cases where TumE performs better than competitors and trying to get what are the features that are actually improves the inference over a standard mixture models. The comparison would be helful also the other way round, showing when the mixture model provides more precise results than TumE. I am wondering if any explanability argument can be raised in these tests.
- In figure 4c how do you explain the error to deviate from normal (ex. 2 picks or skewed distribution)?

**Reply**

Apologies if we misunderstand your question, we believe this is in reference to the two peaks in the 4c % error plot at ~100%. These peaks are generally due to mis-estimates in synthetic samples with either very low mutation rate (e.g. close to 0) or very low frequencies (e.g. ~0.05). In these cases an estimate of 0 is output from the model, hence the error will be 100% since % error is (true - predicted)/true therefore (true-0)/true = 1 or 100%, – also why the density is nearly a spike at this location.
